# Regenerative Endodontic Therapies for Immature Permanent Teeth With Pulp Necrosis: A Review of Biological Foundations, Clinical Outcomes, and Future Frontiers

**DOI:** 10.7759/cureus.115379

**Published:** 2026-08-28

**Authors:** Rishabh Thakur, Arnesh Garg, Ribhav Jha, Hasini Balaji

**Affiliations:** 1 Conservative Dentistry and Endodontics, Sri Ramachandra Institute of Higher Education and Research, Chennai, IND; 2 Oral Medicine and Radiology, Sri Ramachandra Institute of Higher Education and Research, Chennai, IND; 3 Science, Lambert High School, Suwanee, USA; 4 Science, South Forsyth High School, Cumming, USA

**Keywords:** cell-free regenerative endodontic therapy, dentin-pulp regeneration, periapical radiolucency reduction, platelet-rich fibrin (prf) scaffolds, regenerative endodontics, true pulp regeneration

## Abstract

Regenerative endodontics is a very promising biological alternative to root canal treatments because it primarily aims to restore the living pulp-dentin complex instead of just disinfecting and sealing the canal. Current evidence in the field points toward support for symptom relief, periapical healing, continued root development, and apical closure, especially in immature and necrotic teeth. Its core strategies include revascularization, biomaterials, and cell-free methods (exosomes and secretomes). Future directions will most likely have to depend on the improvement of biomaterials, more precise and personalized treatment planning, broader access to stem cells, and stronger translation of research. However, the field’s progress continues to be hindered by barriers such as protocol variability, technique sensitivity, infection control, cost, and limited long-term evidence. As a result, regenerative endodontic therapy should be understood as clinically promising but not yet fully standardized or completely reliable.

## Introduction and background

Regenerative endodontics offers something conventional treatments cannot. It provides a means of restoring living tissue rather than simply removing tissue that is already necrotic. Conventional endodontic methods can eliminate infection but cannot restore the living pulp-dentin complex, and because root development ceases early, treated immature teeth may remain structurally vulnerable [1]. In contrast, regenerative endodontic procedures aim to restore function by promoting the formation of new tissues, continued root maturation, and long-term survival of the tooth [1]. Recent reviews describe the field as a rapidly evolving treatment option that seeks to regenerate the pulp-dentin complex rather than simply maintain a disinfected, empty canal space [2].

This field is clinically important because immature permanent teeth with pulp necrosis pose a unique challenge. Such teeth often present with thin dentinal walls, incomplete root formation, and a high risk of fracture when managed with conventional methods alone [2]. Regenerative strategies are therefore appealing because they may support continued root wall thickening, apical closure, and improved structural integrity of the tooth. Wei et al. [1] established that regenerative endodontic procedures are biologically based and grounded in tissue engineering principles, with stem cells, growth factors, and scaffolds serving as the core components of treatment. Similarly, Murray [2] noted that regenerative approaches are especially valuable for fragile or immature teeth because they may allow continued root development while strengthening the root dentin over time.

The biological basis of the field also helps explain its variable but promising outcomes. In several reports, regenerative treatment has been associated with symptom resolution, increased root length or thickness, and periapical healing [3]. However, the tissue formed within the canal is not "true pulp." Minic et al. [3] found that the newly formed tissue was often fibrous, cementum-like, or bone-like, rather than a fully regenerated pulp-dentin complex. This distinction matters because it implies that many current protocols may promote repair rather than complete regeneration. At the same time, advances in biomaterials, signaling molecules, and stem cell-based methods continue to move the field toward more predictable biological outcomes [1,4].

As such, this review focuses on the biological foundation and clinical potential of regenerative endodontic approaches for immature permanent teeth with pulp necrosis. It also examines the major factors that shape success, including stem cell sources, scaffolds, growth factors, and the clinical settings that influence both treatment selection and outcome. The scope is limited to current regenerative endodontic approaches used in clinical and translational settings, with attention to both the evidence of benefit and the barriers that limit consistent success.

The central research question is: how effective are current regenerative endodontic approaches, and what challenges remain before they can be widely employed? This question is relevant because the field has made substantial progress, yet important limitations persist, including protocol variability, uncertainty about the nature and quality of the tissues generated, and the need for standardized, long-term clinical evidence [1,4].

## Review

Review methodology

This narrative review aims to summarize and recapitulate current evidence about regenerative endodontic therapies for immature permanent teeth with pulp necrosis. Literature was identified and selected through searches of PubMed and Google Scholar. The literature in this review places emphasis on biological mechanisms, clinical outcomes, current limitations, and future directions for regenerative endodontic therapy.

Biological basis

The biological basis of regenerative endodontics starts with the structure and anatomy of the pulp-dentin complex itself. The dental pulp is a soft, living tissue inside the tooth that provides the tooth with nutrition, sensation, immune defense, and the cells required to form the dentin. As the pulp sits inside a hard dentin shell, it has little room to expand when inflamed, which is one reason why pulp injury can become so serious so quickly. This makes regeneration difficult from the start, as a successful treatment must rebuild living tissue in a space that is small, enclosed, and prone to damage [5,6]. Detecting and diagnosing pulpal diseases is a meticulous evaluation process (Figure 1).

**Figure 1:**
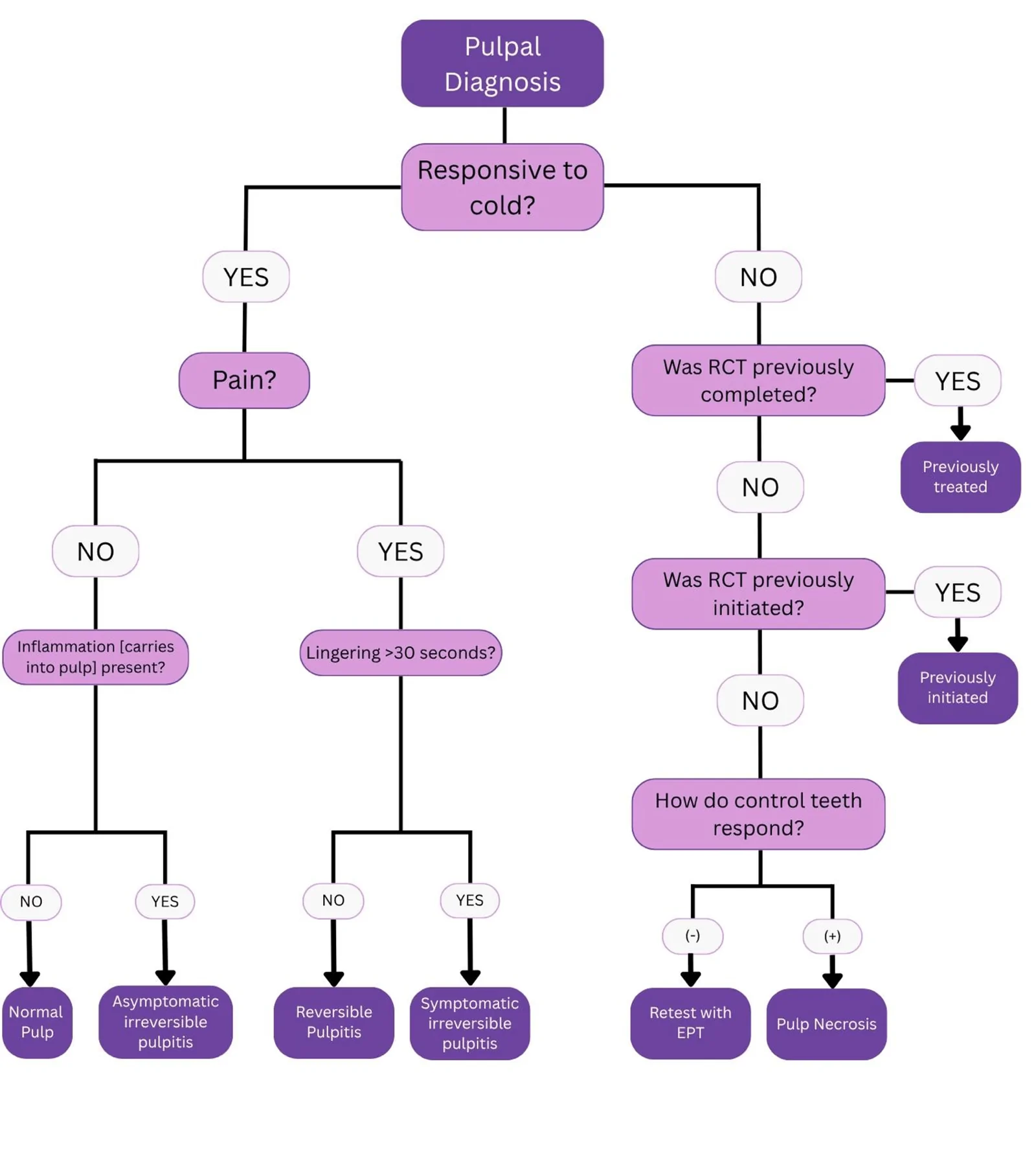
A flowchart depicting the protocol and a stepwise approach to diagnose and manage pulpal diseases and pain RCT, Root Canal Treatment; EPT, Electric Pulp Test. Image credit: Created by the authors using Microsoft Word (Microsoft Corp., Redmond, WA, USA).

Furthermore, the apex of the tooth is the connecting point between the pulp and the other networks of blood supply. Thus, it is also important to examine, evaluate, and treat apical and periapical diseases through a proper protocol and process (Figure 2).

**Figure 2:**
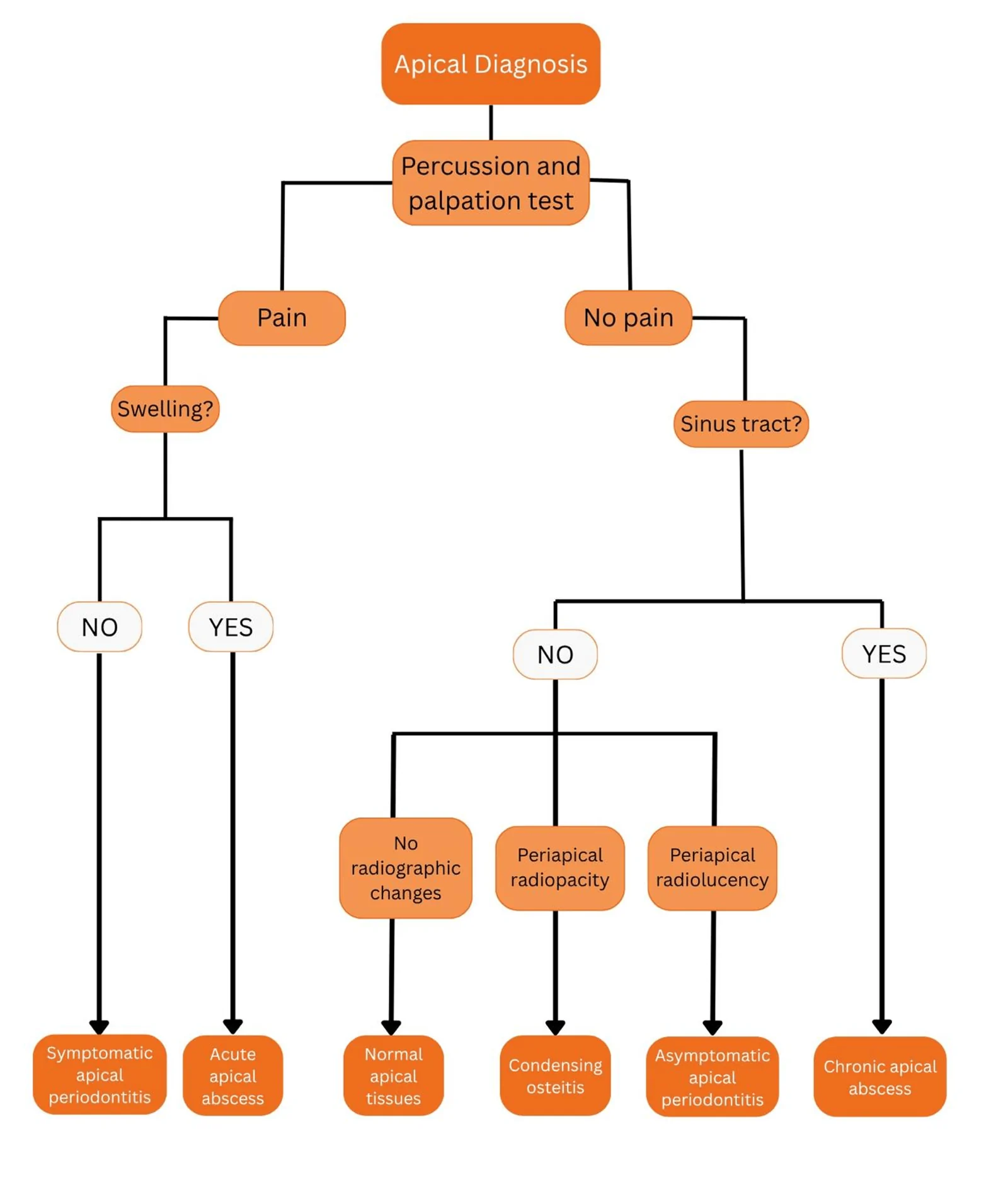
Flowchart depicting the protocol and a stepwise approach to diagnose and manage apical infections, diseases and pain Image credit: Created by the authors using Microsoft Word (Microsoft Corp., Redmond, WA, USA).

Why Regeneration is Difficult

Regenerating the pulp-dentin complex is challenging, as the tooth is not just a hard exterior with a soft center, rather, it is a highly specialized living system. The dental pulp is located inside the dentin, which indicates its limited space to expand when inflamed; the risks within this compact environment are what make infection, trauma, and loss of blood supply extremely dangerous. A tooth that has lost its pulp also loses a lot of its ability to sense injury, continue root development, and to maintain its integrity. Therefore, immature teeth are at special risk, and in that sense endodontic regeneration is not just about filling a canal. It is also about rebuilding a living tissue that can start functioning once more [5,7,8].

Another difficulty is that current regenerative treatment does not always recreate the original pulp. Backly et al. [7] emphasized on the topic of dentin-pulp regeneration, stating that the field still faces major biological challenges and that the real goal is not just to patch damage, but also fully rebuild the complex. Functional regeneration work has shown that the ideal endpoint is recovery of normal/original pulp function, including vascularity, sensitivity, and tissue organization rather than just clinical symptom relief [9]. This distinction is notable as a tooth regeneration treatment may look successful on an X-ray while still containing tissue that is not “true” original pulp. 

Stem Cells and Immune Control

Stem cells are the biological “essentials” of regenerative endodontics, but they work best when the immune system and immune microenvironment are controlled. Li et al. [10] reported that at least six different mesenchymal stem cells (MSC) types have been isolated from postnatal dental tissues, and these cells have remarkable immunomodulatory properties. They also showed that the regenerative and immunomodulatory activity of dental stem cells is shaped by the stem cell/immune microenvironment, which means the local surroundings strongly influence whether healing succeeds or fails. In addition, these cells can suppress inflammation by releasing immunoregulatory cytokines that affect the cells’ innate and adaptive immunity, and inflammatory pulpitis-derived dental pulp stem cells (DPSCs) can suppress macrophage activity through the umor necrosis factor-alpha/indoleamine 2,3-dioxygenase (TNF-α/IDO) axis [10].

DPSCs are very important due to their ability to combine repair potential with their capacity to adapt. Kwack and Lee [5] described DPSCs as MSC-like, highly proliferative, and promising for regenerating damaged dental pulp tissue. They also noted that DPSCs are non immunogenic and may be used in allogeneic transplantation, which makes them especially attractive for future clinical use. Other studies on dental stem cells add that these cells are, in fact, thought to be mesenchymal in origin and can differentiate into osteoblasts, adipocytes, and neural cells, showing just how broad their repair potential may be [8].

Signaling and Repair

Stem cells on their own cannot rebuild the pulp-dentin complex without the right signals. Zhang and Yelick [8] explained that true regeneration depends on the neovascular niche, where DPSCs and MSCs support neurogenesis, angiogenesis, and neovascular inductive activity. That is very significant because the pulp is not just a soft tissue, it is a living sensory and vascular system that must be restored as a whole [11]. Furthermore, it is argued that pulp stem cell-mediated regeneration has convincing evidence for rebuilding the complex histologic structure of native pulp in situ, which is the biological goal that conventional endodontics fails to reach [8]. 

Growth factors and biomaterials help turn this biology into treatment. Brizuela et al. [6] explained that regenerative endodontic therapy relies on the three main tissue engineering elements: stem cells, scaffolds, and growth factors. They also stressed that enhanced neovascularization is needed for more successful complete pulp regeneration, especially in narrow canals where oxygen and nutrient delivery are limited. Hammouda et al. [12] supported this idea by showing that exosomes can promote mesenchymal cell migration, capillary tube formation, Schwann cell behavior, and conversion of pro-inflammatory macrophages into anti-inflammatory ones. Overall, these signaling systems help create a space where repair can inch closer towards true regeneration. 

Clinical Translation and Cell Homing

Regenerative endodontics, from a clinical standpoint, is an emerging treatment line because it offers a biological alternative to treatments that focus only on tooth structure preservation. Yan et al. [13] described cell homing as simpler and more economical than cell-based therapy because it recruits the body’s own cells into the canal rather than transplanting new ones. Their review of recent clinical trials found promising and relevant results, especially in immature teeth, but more mixed outcomes in mature teeth. That makes cell homing one of the most practical current strategies, even though it is still not universally predictable [13].

Other sources further reinforce this same idea. Studies and reviews on dentin pulp regeneration emphasize that the field still faces important challenges and that the real task is to understand how biology and clinical translation can work together better [7]. Literature on inflammation modulation adds that inflammation is not only a cause of necrosis, but is also a prerequisite for neovascularization, stem cell mobilization, and reparative dentin deposition. In other words, successful regeneration depends on balancing the inflammation rather than just removing it [14]. The tissue repair also supports the promise of DPSCs by highlighting their self renewal, paracrine signaling, and immunomodulatory ability [15].

Repair and Regeneration

Many current treatments produce clinical success without fully restoring native pulp tissue. Research on the dentin pulp complex makes it clear that the field is still working toward that deeper goal, while functional pulp regeneration reviews show that true regeneration requires neovascular organization, not just tissue fill in [7,9]. Documented literature regarding broad dental stem cells also reiterate that these cells are flexible repair tools, as they can remain dormant until injury activates them and shifts their role towards differentiation and tissue renewal [8]. Furthermore, the concept of replacing necrotic pulp with inert materials is being challenged by recent advances in pulp biology, which have contributed to an increased number of regenerative strategies rather than those of repair [16]. The key to this is understanding the differences between tissue repair with similar tissues and true natural pulp regeneration (Table 1).

**Table 1:** Comparison of tissue repair and true pulp regeneration

Feature	Repair	Full regeneration
Main goal	Heal the tooth and reduce disease	Regenerate the original pulp-dentin complex
Tissue formed	Fibrous, bone-like/cementum-like	Native pulp-like tissue
Blood vessels	May improve	Must be restored
Sensation	Limited	Ideally returns
Current status	More common	Difficult to achieve consistently

Core regenerative strategies 

The “core” of regenerative endodontics consists of the idea of restoring a living pulp-dentin complex rather than simply taking out infection and sealing the tooth. The main strategies to achieve this include revascularization, stem cell-based repair, scaffolds/biomaterials, growth factors/signaling molecules, and cell-free tactics such as exosomes or secretomes [17-20].

Revascularization and Revitalization

Revascularization, as one of the earliest approaches to regenerative endodontics, relies on blood formation, apical bleeding (following a procedure), and endogenous cells using the canal space to promote healing. This approach is particularly useful because it helps create a biologically flourishing environment. However, it does not guarantee the consistent restoration of “true” pulp tissue. Current reviews also outline that conventional treatments continue to focus on the consequences of pulp exposure rather than the methods to rebuild healthy pulp; this is why revitalization remains as a broader and more demanding goal [18,19]. Revitalization, in that sense, tries to restore the tooth to an almost original state by recovering not only the structure but also activity in the tooth [20,21].

Stem Cells and Secretomes

Stem cells remain important because they provide the cellular source for complete tissue renewal. DPSCs, apical papilla stem cells, and other dental tissue derived stem cells are promising because not only can they generate dental cell lineages, but they can also support tissue healing [19,21]. Concurrently, cell-free strategies are also gaining traction because secretomes and exosomes can provide many similar regenerative signals without requiring the transplant of whole cells. Costa et al. [19] described this particular approach as being based on revascularization and homing induction, while Ahmad et al. [22] showed that dental stem cell derived exosomes can promote odontoblastic differentiation, angiogenesis, inflammation control, and pulp-like tissue formation. Kong et al. [23] also reported that exosomes are promising due to their ability to support proliferation, migration, odontogenesis, angiogenesis, and even neurogenesis; thus, they also may be used in exosome-based regenerative endodontic procedures.

Scaffolds and Biomaterials

Scaffolds are important to regenerative endodontics because they guide cells, retain bioactive molecules, and shape the space where new tissue forms. Hydrogels, in particular, are highly conducive to the field because they can mimic the extracellular matrix (ECM), hold water, transport biomolecules, and carry bioactive agents into the defect site. Furthermore, injectable hydrogels are promising as they are minimally invasive and highly sterile [17]. Other biomaterial reviews make the same points from a broader tissue engineering perspective. Soares et al. [18] noted that biomaterial-mediated pulp engineering requires the balance of safety with ease of use, angiogenesis, mineralization, and functional tissue all while limiting infections and immune reactions.

Furthermore, another review added that polymer scaffolds are extremely important to endodontic regeneration because they carry active molecules and cells into the defect site; they support attachment, proliferation, and angiogenesis [24]. Collagen, gelatin methacrylate (GelMA), and other biodegradable polymers are also described as scaffold materials that mimic the matrix, support cell growth, and enable local delivery of bioactive materials, similar to hydrogels [20]. Hydrogels and synthetic scaffolds, in addition, act as support systems that help stem cells proliferate and differentiate into functional tissue [21].

Growth Factors and Biological Signals

Growth factors and signaling molecules are the basis behind the ability of regenerative endodontics to turn a structural repair idea into a real biological strategy. Once cells enter the canal, they still need guidance as to where to migrate, how to multiply, what to become, and how to organize into tissue that functions like pulp. Machla et al. [25] showed that signaling molecules are central to dentin-pulp regeneration; they also identified basic fibroblast growth factor, vascular endothelial growth factor, bone morphogenetic factor-7, nerve growth factor, and platelet derived growth factor as some of the most studied molecules in this area. These signals are not minor, insignificant add-ons. They influence root development, apical closure, vascularization, innervation, and odontoblast-like cell formation, which makes them a core part of regeneration rather than a supplement to it [25]. In other words, growth factors help determine whether the canal space becomes a biologically empty space or a living tissue environment.

This is notable because the pulp is not a simple tissue. It is a living system with blood vessels, nerves, immune cells, and specialized cells involved in dentin formation and repair. If a regenerative approach does not provide the correct biological signals, the cells that enter the canal may not be able to organize correctly, may fail to survive, more may differentiate into tissues that do not resemble normal pulp. Farjaminejad et al. [20] emphasized this idea by describing regenerative endodontics as dependent on the combined action of polymer scaffolds, stem cells, and growth factors. Furthermore, their work shows that these three engineering tactics are not just a theory. It is instead the practical and realistic foundation of successful regenerative treatment. Growth factors help direct the process towards root development, apical closure, and increased fracture resistance. All of these matter, especially in immature teeth that are structurally fragile [20]. Without these signals, regeneration struggles to occur. 

Furthering the importance of signaling is the fact that different molecules seem to play different roles in different stages of healing. Some signals encourage the cells into the canal, some support angiogenesis, some promote nerve growth, and others encourage the formation of hard tissue. For this reason, regenerative endodontics tends to increasingly depend on carefully designed biological environments rather than one single part of treatment. A review by Machla et al. [25] suggests that the future of pulp regeneration could possibly depend on combining the right molecules in the correct proportions instead of relying on a broad or random signal mix. This idea fits into the larger trend occurring within the field-regeneration is becoming more precise, more controlled, and more biologically targeted.

Prevascularization and Oxygen Supply

A major obstacle in pulp regeneration is keeping the transplanted tissues alive long enough to be able to fully integrate with the surrounding tooth structure. Ruan et al. [26] noted that one of the main difficulties is the engineered tissue’s ability to rapidly vascularize, since avascular pulp tissue would most likely die of ischemia and hypoxia after implantation. Simply put, even when the right cells and materials are present, the tissue continues to fail if it cannot develop a blood supply fast enough. For this reason, prevascularization methods have become increasingly important in pulp regeneration. These methods improve perfusion and oxygenation; they may also include endothelial or endothelial progenitor cells with high angiogenic potential. This is valuable because a regenerated pulp cannot survive without early and sufficient blood supply, no matter how effective the scaffold or stem cells may be [25,26].

Prevascularization is important because not only does it help tissue survive, but it also creates a more realistic healing environment. A regenerated pulp is not just a collection of cells sitting in a canal. It has to become a living, realistic, and functioning tissue with blood flow, oxygen exchange, and the ability to support other forms of repair. If vascularization is delayed, the tissue can break down before it has a chance to organize itself. That is why the topic of blood supply is extremely relevant to regenerative endodontics; the process has to happen quickly enough to prevent cell death, but also in a controlled enough way to allow for proper tissue formation. This is one of many reasons why the field has increasingly moved toward prevascularization as a strategy rather than treating it as “optional” [25,26].

Integrated Strategies

The strongest regenerative strategies combine multiple components at once rather than relying on a single method to do all the work. Farjaminejad et al. [20] explained that regenerative endodontic therapies bring together polymer scaffolds, stem cells, and growth factors, while Atila and Kumaravel [17] showed that hydrogels can also support antimicrobial action and biomolecule delivery. When taken as a whole, these findings show that successful regeneration depends on building a system that can protect the site, direct cell behavior, and keep the environment favorable for healing. A scaffold alone may give structure, but it fails to guide regeneration without biological signals. Stem cells alone may have repair potential, but they need to be paired with a supportive matrix and a supply of signals to organize properly in the canal. 

Ahmad et al. [22] added that exosomes also may work best when used in tandem with scaffolds, growth factors, and endogenous cues. Similarly, Kong et al. [23] reached the conclusion that exosomes are shaped by parental cell type, culture conditions, concentration, and scaffold materials. That means even cell-free strategies are never simple or fully isolated; they still depend on extremely precise designs and the biological environment in which they are used. Moreover, Costa et al. [19] also argued for a more secretome-based sector of regenerative endodontics, especially when the central goal remains to revitalize the pulp while restoring vasculature, nerves, and homeostatic function. Taken together, these studies heavily imply that regeneration works most effectively when multiple treatment systems are combined rather than used individually. 

Overall Strategy

Overall, the core strategies used in regenerative endodontics are not entirely separate ideas but are part of one interconnected system. Revascularization works to bring in blood and cells; stem cell-based therapy provides the ability to regenerate; scaffolds organize the environment; growth factors direct healing; and cell-free methods (such as exosomes and secretomes) offer a more flexible treatment route [17-26].

Clinical evidence and outcomes

Clinical Success and Symptom Relief

Patient-centered outcomes across the available literature converge on a consistent finding: regenerative endodontic therapies achieve high short-term clinical success that appears largely independent of the scaffold employed. In an analysis of 465 immature necrotic teeth, Tang et al. reported failure in only eight teeth, yielding a 98.3% success rate when success was defined as symptom relief and infection control [27]. This material-agnostic pattern is corroborated by Alrashidi et al. who observed comparably high symptom resolution across randomized controlled trials irrespective of whether Platelet-Rich Plasma (PRP), Platelet-Rich Fibrin (PRF), or blood clot scaffolds were used and who attributed outcomes to the overall regenerative approach rather than to any single biomaterial [28]. Evidence from mature teeth extends this reliability beyond the conventional immature-tooth indication: Elheeny et al. reported 95% and 100% success in blood clot and PRF groups, respectively, over 24 months [29], and AlJasser et al. documented uneventful healing of combined endodontic-periodontal lesions without adverse events [30]. Taken together, and consistent with reports that regenerative treatment of mature teeth yields clinical and radiographic outcomes comparable to non-surgical root canal therapy [29], these findings suggest that reliability derives from the biological environment established rather than the choice of scaffold.

These conclusions warrant caution. Pooled success rates, as in Tang et al. aggregated heterogeneous protocols, follow-up intervals, and outcome definitions, obscuring between-study variation [27]. The mature-tooth findings rest on the weakest evidence: the small sample of Elheeny et al. yielded wide confidence intervals in which a 100% success rate was highly sensitive to individual failures, while AlJasser et al. provided case-level rather than comparative data [29,30]. Follow-up was generally limited to 24 months or less, outcomes were clinician-assessed rather than blinded, and radiographic healing served only as a surrogate endpoint. Critically, symptom relief, low reinfection rates, and radiographic resolution reflected the absence of clinical failure rather than confirmed pulp regeneration; histological verification remained rare, and short-term data could not establish long-term tooth survival, continued root development, or restored vitality. Regenerative endodontics may therefore be regarded as clinically reliable for symptom and infection control, tentatively including mature teeth, though claims of true regeneration and durability exceed the current evidence.

Radiographic Healing and Periapical Lesion Reduction

Radiographic healing provides a second, more biologically meaningful axis of reliability, complementing symptom relief by indicating resolution of periapical disease rather than mere symptom suppression. The evidence suggests a scaffold-dependent pattern that is most pronounced early and attenuates over time. Tang et al. found that platelet concentrates conferred a modest advantage, with PRP producing superior periapical healing relative to blood clot in one subgroup (relative risk 1.13; P=0.03) [27]. This temporal dimension was clarified by Elheeny et al. who tracked periapical index (PAI) scores at three, six, 12, and 24 months and observed that PRF may accelerate early radiographic healing without conferring a durable long-term advantage over a blood clot [29]. Alrashidi et al. situate these findings within randomized controlled trials, treating reduction of periapical radiolucency as a primary outcome and thereby strengthening the credibility of the observed healing while noting persistent variability across scaffold types [28]. Together, the literature indicates that platelet concentrates may hasten periapical resolution in the short term, but that this benefit converges with blood clot outcomes as follow-up lengthens.

Several limitations temper these conclusions. The scaffold effect reported by Tang et al. was derived from a single subgroup with a relative risk of only 1.13, a small effect size whose statistical significance may reflect large pooled samples more than clinical magnitude, and subgroup analyses are inherently prone to false-positive findings [27]. Elheeny et al. contributed valuable longitudinal data but was constrained by a small sample, limiting the precision of its periapical index comparisons [29]. More fundamentally, the studies resisted direct comparison owing to heterogeneous imaging modalities, follow-up intervals, and scaffold groupings, and the PAI itself is an ordinal, observer-dependent measure susceptible to interpretation bias, particularly where radiographic assessment is unblinded. Clinically, these data support periapical healing as a genuine and reproducible outcome of regenerative endodontic therapy, but they do not establish the superiority of any single scaffold. The transient early advantage of platelet concentrates is unlikely to justify preferential selection when longer-term radiographic outcomes appear equivalent.

Root Maturation and Apical Closure 

Root maturation and apical closure represent the most biologically demanding measures of regenerative success, since they test whether these therapies can restore continued development rather than merely arrest diseases. In immature necrotic teeth, meaningful maturation is evidenced by increased root length, thickening of dentinal walls, and narrowing or closure of the open apex-outcomes that distinguish true regeneration from the passive apical barrier produced by apexification. Panda et al. articulated this distinction, positioning regenerative endodontic procedures as clinically superior to apexification precisely because they aim to support ongoing root development rather than only seal the apex, thereby conferring a potentially greater long-term structural benefit [31]. Widbiller et al. supplied quantitative support through a cell-transplantation study reporting root lengthening of 4.32 to 6.16 mm and pronounced apical narrowing within 12 months, exceeding that achieved by apexification [32]. Yan et al. complemented these transplantation data by reviewing cell-homing trials, which suggested that comparable regeneration may arise through recruitment of the patient’s own progenitor cells into the canal space rather than exogenous cell delivery [13]. Considered together, the literature indicates that regenerative procedures can drive measurable, developmentally relevant changes in root structure, positioning root maturation as among the most compelling indicators of genuine regeneration.

Nonetheless, these findings remain preliminary. The tissue-engineering evidence and the cell-transplantation data of Widbiller et al. [32] in particular was derived from small samples with follow-up limited to roughly 12 months, insufficient to establish that early root lengthening translates into durable maturation or long-term tooth survival. Small cohorts widened confidence intervals and heightened susceptibility to selection bias, while the technical complexity and cost of cell-based protocols further constrained generalizability to routine practice. The cell-homing paradigm reviewed by Yan et al., though more clinically translatable, likewise rested on heterogeneous trials of variable rigor [13]. Clinically, these data justify cautious optimism: regenerative procedures appear capable of promoting continued root development in a way apexification cannot, which is meaningful for the structural prognosis of immature teeth, but the evidence is best characterized as promising rather than definitive. Larger controlled trials with extended follow-up are required before continued maturation can be regarded as a reliably reproducible outcome.

Scaffold Comparisons and Treatment Reliability

Scaffold selection constitutes a further axis for evaluating regenerative outcomes, on the premise that the material supporting tissue ingrowth may itself shape healing and root development. Across the available comparisons, platelet concentrates, and PRF in particular, emerged as the leading candidates, though the evidence stopped short of establishing a definitive hierarchy. Narang et al. compared blood clot, PRP, and PRF in immature necrotic permanent teeth, and found PRF most capable of catalyzing regenerative growth, suggesting it may provide a more favorable biological environment in at least some cases [33], a pattern echoed by reports that PRP and PRF yield the most favorable short-term healing among available scaffolds [34]. Markandey et al. contributed methodological rigor to the same three-way comparison by combining clinical assessment with intraoral periapical radiography and cone beam computed tomography (CBCT), underscoring that scaffold reliability should be judged not by symptom relief alone but by structural healing visible on imaging [35]. Kawthar et al. extended the comparison beyond the conventional triad, evaluating PRF against concentrated growth factor in mature necrotic teeth and signaling a move toward newer platelet-derived biomaterials [36]. Taken together, these studies indicated that scaffold choice may influence regenerative outcomes, with PRF the most consistently promising option for supporting early healing and growth.

This apparent advantage should be interpreted conservatively. Much of the support for PRF rests on single studies with small samples, from which a consistent superiority cannot be inferred; small cohorts widen confidence intervals and are especially vulnerable to selection bias and to the absence of blinding in outcome assessment. Methodological heterogeneity compounded the problem, as the studies differed in imaging modalities, outcome definitions, and - in the case of Kawthar et al. - tooth maturity and comparator scaffold, precluded pooled or head-to-head interpretation [36]. The stronger imaging-based designs, such as the CBCT assessment of Markandey et al. improved internal validity but remained limited by short follow-up [35]. Clinically, the evidence supports PRF as a reasonable and biologically favorable scaffold choice and justifies continued investigation of emerging platelet-derived materials, but it does not warrant designating any single scaffold as superior; scaffold selection may therefore remain guided as much by availability, cost, and operator familiarity as by demonstrated regenerative advantage until larger standardized trials are conducted.

Follow-Up, Long-Term Survival, and Evidence Limitations

To further evaluate whether regenerative endodontic therapies are credible beyond short-term clinical improvement, follow-ups and long-term survival rates are essential. Mujawar et al. directly addressed this issue by comparing traditional root canal therapy in permanent teeth with periapical lesions to regenerative endodontic procedures [37]. This comparison was especially important because regenerative procedures may show promising healing, but they must still be judged against conventional root canal treatment, remaining as the more established method of care; however, root canal treatment removes pulpal vitality, which can weaken the tooth’s long-term structural configuration [37]. Furthermore, regenerative procedures provided evidence for comparable or even superior clinical and radiographic healing results compared to conventional treatments [37]. A systematic review found that regenerative endodontic procedures improved pulp vitality responses and supported ongoing tissue healing in teeth with endodontic periapical lesions [36]. It was also reported that all clinical trials found significant evidence for periapical lesion-size reduction over time [36]. Brizuela et al.’s trial found a 100% tooth survival at 12 months after treatment without any major adverse effects [6]. Within the same 12-month time period, regenerative endodontic procedures and traditional root canal treatments had comparable success rates, having a pooled odds ratio of 0.57 and no statistically significant difference [6]. Additionally, in a 36 month clinical trial, regenerative endodontic procedures performed with either calcium hydroxide or modified triple antibiotic paste showed high survival and success rates, with radiographic improvements in both groups [38]. This aids in reaching a common conclusion that regenerative endodontic procedures may be clinically reliable alternatives to conventional treatments in mature, permanent teeth - especially when seen in the perspective of tooth retention, periapical healing, and absence of complications. Although results and outcomes seemed positive, Mujawar et al. mentioned that evidence was not fully definitive because the overall quality was moderate and limited by small sample sizes, differences in methodology, and the necessity of multicenter trials with longer follow-up periods [37]. 

Limitations and challenges

Biological Limitations: Stem Cell Survival and Differentiation

Although regenerative endodontic treatments yield positive clinical outcomes, their dependability is limited by the true reality of what happens to stem cells inside the canal space. On paper, stem cells should survive, migrate into the root canal, react to scaffold and growth-factor signals, and further differentiate into pulp-like cells that help to form a coherent tissue. Junaid et al. [39] however, found that stem-cell regeneration is not as simple of a process as blatantly placing cells into a tooth and expecting pulp tissue to grow. The study explained that major challenges still existed, including stem-cell differentiation, immune responses, long-term safety, the best scaffold materials for cell delivery, the source and separation of stem cells, and the challenges in guiding stem cells to become the correct cell types within the pulp environment. Even when regenerative procedures may seem clinically successful, the biological processes behind that success may still be uncertain. Chen et al. [40] contributed to this matter by adding that cell-based pulp regeneration is threatened by low cell survival and immune rejection, particularly when cells are placed in an environment that houses inflammation, hypoxia, or infections. In these stressful conditions, there is a lot at risk: stem cells may die, fail to attach, fail to differentiate correctly, or produce disjointed repair-like tissue instead of the true, expected pulp tissue. Further, Beco et al. [41] have claimed that blood clot formation does not immediately correlate to organized pulp regeneration. As a result, even the strongest of clinical outcomes may not always show complete pulp regeneration, because the role and behavior of stem cells inside the canal is, at this moment, beyond one’s control. 

Clinical and Protocol Challenges

Because protocols and procedures have not yet been standardized amongst labs, regenerative endodontic treatments remain uncertain and limited. Even though stem cells have regenerative potential, success depends heavily on how the treatment is performed, such as which scaffold was used, which growth factors were applied, how the canal was disinfected, and how often the patient was followed-up after treatment. Alshamrani et al. [42] mentioned that treatments of this sort remain limited due to small sample sizes, protocol heterogeneity, and limited follow-ups. All these factors together make it difficult to judge the predictability of these treatments in large patient populations. Beco et al. [41] further added that clinicians may not always know which patients are the ideal candidates or which protocol will produce an optimal outcome. Together, these complications help find that regenerative treatments can be clinically promising, however, they still remain uncertain due to differences in protocol and treatment characteristics. 

Technique Sensitivity and Infection Control

Technique sensitivity is also another reason regenerative endodontic treatments can be limited, specifically when infection control is involved. Regenerative endodontic procedures require disinfecting the tooth while simultaneously attempting to create an environment that supports stem cell activity, scaffold function, and tissue repair. This process differs heavily from conventional root canal treatments, where the main goal is to sterilize and seal the canal. According to Dos Reis-Prado et al. [43], cleaning and disinfecting immature teeth is regarded as challenging because bacteria can infiltrate deeply into dentinal tubules and other areas that are inaccessible. These challenges pose a major issue because any remaining bacteria can impede the healing process and further elevate the risk of persistent infections. However, treatments cannot solely rely on aggressive disinfection as the procedure also needs to protect the preexisting biological conditions required to accommodate stem cells and growth factors. This concern is held by Junaid et al. [39] as they noted that treatment success depends on optimizing biomaterials, delivery systems, and growth factor administration. All in all, regenerative endodontic techniques are not simply a matter of placing regenerative materials into the canal, they requires precise clinical technique, meticulous disinfection, and regulated biological conditions. 

Practical, Ethical, and Translational Barriers

Because of the many rules and regulations, regenerative endodontics also faces practical and ethical challenges, affecting whether these therapies can move forward from a research setting into an everyday dental clinic. Xie et al. [9] explicated that personalized cell therapy using self-derived DPSCs or stem cells from exfoliated deciduous teeth can be difficult to replicate into a clinical practice because of its need for a GMP laboratory resulting in high costs to maintain and order. Stem-cell based treatments are not simply an intraoral procedure; they require cell isolation, expansion, storage, quality assurance, and specific equipment and dental tools. Alsharami et al. [42] have explained that autologous stem cells may require surgical intervention, while allogeneic stem-cell procedures may foster autoimmune conditions. Beco et al. [41] have said that ex vivo cell manipulation can involve cross-contamination risks, autoimmune conditions, regulatory barriers, and high costs. These issues not only create safety concerns but also ethical ones. Clinicians must consider whether regeneration is possible as well as whether it can be delivered safely, affordably, and consistently. The challenge is not only biological success, but also ensuring regenerative endodontic treatments are realistic, safe, and widely accessible for patients. 

Final Evaluation: Promising but not Fully Established

Overall, regenerative endodontics remain promising, but its credibility is still limited by biological uncertainty, inconsistent clinical protocols, technique sensitivity, practical barriers, and limited long-term evidence. Until standardized methods, larger human trials, and longer follow-up periods become available, these treatments should be seen as developing rather than casual, everyday dental practices. 

Future directions

Improved Biomaterials and Scaffold Design

The advancements in biomaterials and scaffold design greatly impact the future of regenerative endodontics. In current regenerative procedures, treatments rely on scaffolds to support cell migration, tissue organization, growth factor delivery, and healing inside the canal space. However, the outcomes produced by these scaffolds are highly inconsistent. Thus, future materials need to become more predictable and biologically active. New biomaterials such as gelatin, chitosan, alginate, and DNA-based nanomaterials could possibly improve biocompatibility, antibacterial performance, and regenerative potential beyond traditional calcium-based cements [44]. Farjaminejad et al. have explained that new emerging technologies, such as multifunctional scaffolds, nanotechnology, bioactive components, and smart drug-delivery systems, have the potential to improve antimicrobial activity and facilitate coherent tissue interactions [20]. Successful scaffolds should not only be able to hold cells in place, but also help minimize infections, release signals, and facilitate tissue formation [20]. Alothman et al. further emphasized that advances in biomimetic scaffolds and growth factors delivery methods are promising, however a highly centralized clinical protocol is necessary [4]. Once viewed together, the evidence offers new directions in the future of scaffold design and improved clinical consistency. 

Precision and Personalization 

Personalized regenerative endodontic treatments are another major future direction. Instead of following a general protocol for all patients, future therapies may accommodate therapies that are personalized with each patient. These personalizations will be determined from tooth maturity, infection severity, root anatomy, patient age, available stem cell sources, and healing potential. Farjaminejad et al. found that personalized medicine and patient-specific scaffolding were promising directions for the future of endodontic treatments [20]. Saberian et al. explained that artificial intelligence has the potential to help customize treatment plans per imaging, genomic data, and patient records to improve diagnostic accuracy and predict treatment effectiveness [45]. Through these methods, precision-based care could help clinicians choose the most appropriate scaffold, regenerative strategy, or growth factor system for each unique case rather than relying on a standardized method.

Improving Stem Cell Access and Research Translation

Another significant future direction in regenerative endodontics is making stem cells easily accessible while also improving the speed of translation from clinical trial results to usable clinical treatment. Because regenerative endodontics still faces important scientific, regulatory, and commercialization hurdles, cell-free approaches that rely on biomaterial scaffolds and growth factors to recruit the body’s own cells may help move the field toward clinical use more efficiently [46]. Tooth banking may also become very important here since dental stem cells can be preserved for later use while still retaining the ability to differentiate into odontogenic, neurogenic, and adipogenic types [47]. At the same time, exosome-based strategies can help shrink the gap between cell biology and clinical treatment because engineered exosomes can be modified or tweaked for better targeting/therapeutic performance; this occurs while the exosomes continue to support possibilities for diagnosis and therapeutic performance in a more controlled way. This kind of approach could reduce dependence on complex cell handling while simultaneously preserving potential for regeneration [48]. To further advance the field, researchers will also need stronger legal, ethical, and standardized research frameworks so that stem cell therapies can become more safe, more reliable, and more consistent in real clinical settings [47,48]. 

Research Priorities for the Field

All in all, the leading research priority is to initially make regenerative endodontics more standardized and evidence-based. Farjaminejad et al., suggested that studies should include randomized controlled clinical trials with follow-up periods of a minimum of 24 to 36 months [20]. Saberian et al. also mentioned the need for standardized and precise databases that contain different patients and clinical environments [44]. Early clinical use should be ordered through supervised translation pathways with risk tiered review, standardized processing, and sufficient outcome capture so that early cases produce understandable evidence [49]. Overall, future directions should branch onto larger clinical trials, longer follow-up periods, protocol standardization, improved biomaterials, and the introduction of personalized treatment planning. These stabilizations would help regenerative endodontic treatments to move from a promising experiment toward a more reliable practice.

## Conclusions

Regenerative endodontics occupies a position of substantial promise tempered by unresolved questions of predictability. Its distinctive value lies in a biological ambition that conventional therapy cannot match: whereas root canal treatment disinfects and seals the canal at the cost of pulpal vitality, regenerative procedures aim to restore the living pulp-dentin complex and, in immature necrotic teeth, to sustain continued root development, apical closure, and dentinal wall thickening alongside symptom relief and periapical healing. This developmental capacity is the field’s clearest clinical advantage and the reason its outcomes cannot be judged by symptom control alone. The biological toolkit underlying these gains - stem cells, scaffolds, growth factors, revascularization, and emerging cell-free strategies such as exosomes - continues to expand, and anticipated advances in biomimetic scaffolds, controlled growth-factor delivery, 3D printing, and artificial-intelligence-guided treatment planning offer a plausible route toward greater precision and consistency.

The central limitation, however, is that current success largely reflects repair-like healing rather than confirmed pulp regeneration. Across the evidence base, favorable outcomes are constrained by small sample sizes that widen uncertainty, by protocol and technique variability that impede reproducibility, and by short follow-up and unblinded, surrogate-based assessment that heighten the risk of bias and leave long-term survival unestablished. Practical barriers such as cost, training demands, and translational complexity further limit generalizability to routine practice. Regenerative endodontic therapies should therefore be regarded as clinically promising rather than established alternatives to conventional treatment: appropriate for carefully selected cases, particularly immature necrotic teeth, but not yet supported by evidence sufficient to displace root canal therapy as standard care. Realizing the field’s potential will require larger multicenter human trials, standardized protocols, longer follow-up, histological confirmation of regenerated tissue, and more accessible technologies before predictability can match the biological promise.

## References

[1] Wei X, Yang M, Yue L (2022). Expert consensus on regenerative endodontic procedures. Int J Oral Sci.

[2] Murray PE (2023). Review of guidance for the selection of regenerative endodontics, apexogenesis, apexification, pulpotomy, and other endodontic treatments for immature permanent teeth. Int Endod J.

[3] Minic S, Vital S, Chaussain C, Boukpessi T, Mangione F (2022). Tissue characteristics in endodontic regeneration: a systematic review. Int J Mol Sci.

[4] Alothman FA, Hakami LS, Alnasser A, AlGhamdi FM, Alamri AA, Almutairii BM (2024). Recent advances in regenerative endodontics: a review of current techniques and future directions. Cureus.

[5] Kwack KH, Lee HW (2022). Clinical potential of dental pulp stem cells in pulp regeneration: current endodontic progress and future perspectives. Front Cell Dev Biol.

[6] Brizuela C, Huang GT, Diogenes A, Botero T, Khoury M (2022). The four pillars for successful regenerative therapy in endodontics: stem cells, biomaterials, growth factors, and their synergistic interactions. Stem Cells Int.

[7] El Backly R, Zaky S, Marei MK (2023). Editorial: Regenerating the dentin-pulp complex: understanding the challenges that lie ahead. Front Bioeng Biotechnol.

[8] Zhang W, Yelick PC (2021). Tooth repair and regeneration: potential of dental stem cells. Trends Mol Med.

[9] Xie Z, Shen Z, Zhan P (2021). Functional dental pulp regeneration: basic research and clinical translation. Int J Mol Sci.

[10] Li P, Ou Q, Shi S, Shao C (2023). Immunomodulatory properties of mesenchymal stem cells/dental stem cells and their therapeutic applications. Cell Mol Immunol.

[11] Shi X, Hu X, Jiang N, Mao J (2025). Regenerative endodontic therapy: from laboratory bench to clinical practice. J Adv Res.

[12] Hammouda DA, Mansour AM, Saeed MA, Zaher AR, Grawish ME (2023). Stem cell-derived exosomes for dentin-pulp complex regeneration: a mini-review. Restor Dent Endod.

[13] Yan H, De Deus G, Kristoffersen IM (2023). Regenerative endodontics by cell homing: a review of recent clinical trials. J Endod.

[14] Zaky SH, Shehabeldin M, Ray H, Sfeir C (2021). The role of inflammation modulation in dental pulp regeneration. Eur Cell Mater.

[15] He J, Fu J, Wang R (2025). Tissue repair mechanisms of dental pulp stem cells: a comprehensive review from cutaneous regeneration to mucosal healing. Curr Issues Mol Biol.

[16] Zhou C, Yuan Z, Xu H, Wu L, Xie C, Liu J (2022). Regenerative endodontic procedures in immature permanent teeth with dental trauma: current approaches and challenges. Front Dent Med.

[17] Atila D, Kumaravel V (2023). Advances in antimicrobial hydrogels for dental tissue engineering: regenerative strategies for endodontics and periodontics. Biomater Sci.

[18] Soares DG, Bordini EA, Swanson WB, de Souza Costa CA, Bottino MC (2021). Platform technologies for regenerative endodontics from multifunctional biomaterials to tooth-on-a-chip strategies. Clin Oral Investig.

[19] Costa LA, Eiro N, Vaca A, Vizoso FJ (2022). Towards a new concept of regenerative endodontics based on mesenchymal stem cell-derived secretomes products. Bioengineering (Basel).

[20] Farjaminejad R, Farjaminejad S, Garcia-Godoy F (2025). Regenerative endodontic therapies: harnessing stem cells, scaffolds, and growth factors. Polymers (Basel).

[21] Umapathy VR, Natarajan PM, Swamikannu B (2025). Regenerative strategies in dentistry: harnessing stem cells, biomaterials and bioactive materials for tissue repair. Biomolecules.

[22] Ahmad P, Estrin N, Farshidfar N, Zhang Y, Miron RJ (2025). Mechanistic insights into dental stem cells-derived exosomes in regenerative endodontics. Int Endod J.

[23] Kong Q, Wang Y, Jiang N (2024). Exosomes as promising therapeutic tools for regenerative endodontic therapy. Biomolecules.

[24] Sugiaman VK, Jeffrey Jeffrey, Naliani S, Pranata N, Djuanda R, Saputri RI (2023). Polymeric scaffolds used in dental pulp regeneration by tissue engineering approach. Polymers (Basel).

[25] Machla F, Angelopoulos I, Epple M, Chatzinikolaidou M, Bakopoulou A (2022). Biomolecule-mediated therapeutics of the dentin-pulp complex: a systematic review. Biomolecules.

[26] Ruan Q, Tan S, Guo L, Ma D, Wen J (2023). Prevascularization techniques for dental pulp regeneration: potential cell sources, intercellular communication and construction strategies. Front Bioeng Biotechnol.

[27] Tang Q, Jin H, Lin S, Ma L, Tian T, Qin X (2022). Are platelet concentrate scaffolds superior to traditional blood clot scaffolds in regeneration therapy of necrotic immature permanent teeth? A systematic review and meta-analysis. BMC Oral Health.

[28] Alrashidi A, Sadaf D, Alabdulrazaq R, Alrashidi A, Alajlan M, Aljuhani A (2021). Effectiveness of various scaffolds on the success of endodontic tissue regeneration: a systematic review and meta-analysis of randomized controlled trials. Saudi Endod J.

[29] Elheeny AA, El Bahnasy SS, ElMakawi YM, Turky M, Ahmed EF, Wahba NK (2025). Clinical and radiographic outcomes of non-surgical retreatment of mature maxillary incisions using two regenerative endodontic techniques in adolescents: a 24-month randomized clinical trial. BDJ Open.

[30] AlJasser R, Bukhary S, AlSarhan M, Alotaibi D, AlOraini S, Habib SR (2021). Regenerative therapy modality for treatment of true combined endodontic-periodontal lesions: a randomized controlled clinical trial. Int J Environ Res Public Health.

[31] Panda P, Mishra L, Govind S, Panda S, Lapinska B (2022). Clinical outcome and comparison of regenerative and apexification intervention in young immature necrotic teeth: a systematic review and meta-analysis. J Clin Med.

[32] Widbiller M, Knüttel H, Meschi N, Durán-Sindreu Terol F (2023). Effectiveness of endodontic tissue engineering in treatment of apical periodontitis: a systematic review. Int Endod J.

[33] Narang I, Mittal N, Mishra N (2015). A comparative evaluation of the blood clot, platelet-rich plasma, and platelet-rich fibrin in regeneration of necrotic immature permanent teeth: a clinical study. Contemp Clin Dent.

[34] Sabeti M, Ghobrial D, Zanjir M, da Costa BR, Young Y, Azarpazhooh A (2024). Treatment outcomes of regenerative endodontic therapy in immature permanent teeth with pulpal necrosis: a systematic review and network meta-analysis. Int Endod J.

[35] Markandey S, Das Adhikari H (2022). Evaluation of blood clot, platelet-rich plasma, and platelet-rich fibrin-mediated regenerative endodontic procedures in teeth with periapical pathology: a CBCT study. Restor Dent Endod.

[36] Kawthar BS, Fatma B, Hanen B, Imen G, Souha BY (2025). Regenerative endodontics in the treatment of periapical lesions of endodontic origin: a systematic review of randomized controlled trials. J Contemp Dent Pract.

[37] Mujawar A, Pandit V, Shaikh S (2025). Comparative outcomes and success rates of regenerative endodontics versus traditional root canal therapy: a systematic review and meta-analysis. J Int Med Res.

[38] Al-Qudah A, Almomani M, Hassoneh L, Awawdeh L (2023). Outcome of regenerative endodontic procedures in nonvital immature permanent teeth using 2 intracanal medications: a prospective randomized clinical study. J Endod.

[39] Junaid M, Alyami S, Alkayraan FS (2025). Advancements in regenerative endodontics: a systematic literature review of stem cell-based therapies, in vitro and in vivo evidence, clinical relevance, and future prospects. J Pioneering Med Sci.

[40] Chen Y, Hu M, Lee YL (2026). Progress and perspectives in allogeneic cell-based pulp regeneration. Int Endod J.

[41] Beco M, Di Pasquale F, Valenti C (2026). Pulp-dentin regeneration via cell homing: current evidence and perspectives on cell-free regenerative endodontic therapy. Medicina (Kaunas).

[42] Alshamrani WA, Alzarea SS, Alabbas JK, Alabbas AK, Aljaddua MI, Khattak O, Issrani R (2026). Regenerative endodontics and stem cell-based therapies - a systematic review. J Pharm Bioallied Sci.

[43] Dos Reis-Prado AH, Benetti F, Bottino MC, Dal-Fabbro R (2025). Optimizing methods for regenerative endodontics. Regen Med.

[44] Rajasekar V, Abdalla MM, Huang M, Neelakantan P, Yiu CK (2025). Next-generation biomaterials for vital pulp therapy: exploring biological properties and dentin regeneration mechanisms. Bioengineering (Basel).

[45] Saberian E, Jenča A, Jenča A, Zare-Zardini H, Araghi M, Petrášová A, Jenčová J (2024). Applications of artificial intelligence in regenerative dentistry: promoting stem cell therapy and the scaffold development. Front Cell Dev Biol.

[46] Mao JJ, Kim SG, Zhou J (2012). Regenerative endodontics: barriers and strategies for clinical translation. Dent Clin North Am.

[47] Chintha V, Gantala R, Katne T, Anees K, Janumpally VT, Poreddy VR (2021). Stem cell therapy in oral mucosal lesions: a future vision. Int J Sci Res.

[48] Ren J, Jing X, Liu Y (2023). Exosome-based engineering strategies for the diagnosis and treatment of oral and maxillofacial diseases. J Nanobiotechnology.

[49] Nakashima M, Kobayashi E (2026). Beyond the case series: a supervised translation pathway for regenerative endodontics. Res Connect.

